# Simple pH-Triggered Control over Hydrogel Formation by Acetyl Valine

**DOI:** 10.3390/molecules30163345

**Published:** 2025-08-11

**Authors:** Roberta Stile, Devis Montroni, Demetra Giuri, Claudia Tomasini

**Affiliations:** Dipartimento di Chimica Giacomo Ciamician, Università di Bologna, Via Piero Gobetti, 85, 40129 Bologna, Italy; roberta.stile2@unibo.it (R.S.); devis.montroni@unibo.it (D.M.); demetra.giuri2@unibo.it (D.G.)

**Keywords:** Ac-Val, hydrogels, pH change, self-assembly, trigger

## Abstract

This paper reports on the use of acetyl-L-valine (Ac-Val) as an effective and precise pH modifier for inducing hydrogel formation. Ac-Val offers several advantages: it is fully water-soluble, overcoming dissolution issues, and allows for stock solution preparation to fine-tune trigger volume and final material pH. As a weaker carboxylic acid compared to inorganic acids, Ac-Val enables more controlled pH variation. For comparison, a commercial lactic acid (LA) solution was also evaluated. The reliability of Ac-Val as a pH modifier was tested on three amino acid derivatives—Boc-Dopa(Bn)_2_-OH, Lau-Dopa(Bn)_2_-OH, and Pal-Phe-OH, all known to be efficient gelators. These molecules, sharing common structural features, form gels varying in transparency, robustness, and elasticity. Notably, Pal-Phe-OH is a supergelator. A key benefit of Ac-Val lies in its ability to cause an instantaneous pH modification, allowing for precise pH adjustment before the gel network forms. This pH-change approach with Ac-Val demonstrates broad applicability, enabling the creation of gels with tailored pH values for various acidic molecules, which is particularly valuable for applications like drug delivery where specific pH environments are crucial.

## 1. Introduction

The aim of this work is to envision a straightforward, tunable, and easily reproducible technique to prepare hydrogels at defined pH values, with a particular focus on biocompatible hydrogels, specifically those within the physiological pH range around 7. This aspect is of paramount importance when the hydrogel is intended for biological applications, such as drug delivery systems. Such applications invariably require a well-defined pH environment, which may exhibit considerable variability depending on the specific target tissue and the intended application [1]. Indeed, it is crucial to acknowledge the heterogeneity of pH values across various anatomical locations within the body [2], including substantial alterations in the baseline pH due to the presence of tumors and infections [3,4,5]. Finally, pH may highly impact the drug release [6,7,8].

Low-molecular-weight gelators (LMWGs) offer a wide tunability of the chemical structure and of the conditions necessary for the gelation process. These compounds form supramolecular structures thanks to non-covalent interactions between aromatic rings, proton donors and acceptors, and hydrophobic moieties [9,10,11,12,13]. LMWGs are often designed to respond to specific triggers, such as ultrasound sonication, addition of a non-solvent or of salts, pH changes, and enzymes [14,15,16,17,18,19]. When the LMWG is an amino acid derivative, a common strategy is to exploit its free carboxylic group—the addition of an acid to the alkaline solution of the gelator will trigger gel formation [19]. This reversible sol/gel process driven by the pH may be exploited for drug delivery purposes [8,20,21,22,23,24].

A fast drug release may be desirable for some applications, and this can be achieved with a trigger that disassembles the gel at a specific pH or with highly soft materials with poor mechanical properties [25]. Physical gels, like those obtained with LMWGs, are also degraded over time by the physiological environment. This process leads to the disassembly of the fibers [26], held together by non-covalent interactions, with the release of the native gelator. This is the reason why it is usually preferable to perform the biocompatibility essay on the gelator molecules [12,27] in addition to that on the gel as a material. A possible strategy to reduce the degradation kinetics and ensure a higher retention of the gel form, apart from the introduction of crosslinking, may be to increase the gel concentration.

To this end, identifying a suitable gelator is undoubtedly crucial, but the selection of an appropriate pH modifier is equally important. Indeed, if the pH reduction is achieved by the addition of a strong acid, such as hydrochloric acid [28], the pH is immediately altered with the rapid formation of the protonated form of the gelator, which is insoluble in water. This excessively fast transformation leads to the formation of aggregates or precipitates, hindering the creation of a homogeneous fibrous network. The formation of a gel, suitable for subsequent applications or modifications, requires a sufficient amount of time for the creation of fibrils that self-assemble into fibers, trapping the solvent in the process.

A valuable alternative to this process was proposed by Adams [29], who suggested the use of δ-gluconolactone (GdL) as a slow pH modifier. Indeed, the hydrolysis of GdL yields gluconic acid, which reduces the pH of the solution over time, allowing the sample to achieve a uniform pH and promoting the slow formation of fibrils through the self-aggregation of the insoluble acid. However, with this method the final pH is reached after several hours, by which time the gel has already formed. Furthermore, GdL is a solid and, upon addition, requires a certain period for dissolution, which can affect the homogeneity of the final gel. Inspired by the same hydrolysis concept of GdL, the same group also proposed organic anhydrides as possible alternatives to slowly trigger the pH shift [30].

Another strategy involves the use of buffer solutions at defined pH ranges, but this approach suffers from the presence of inorganic salts in the solution, which could interfere with the self-aggregation process of the gelator and alter the properties of the final gel [31].

In a previous work, we established that free amino acids could serve as triggers for gel formation, yielding gels with distinct final pH values determined by the amino acid selected [31]. Expanding on our understanding of gelation triggers, we have also reported the use of Boc-L-Ala-Aib-L-Val-OH, a tripeptide with a free carboxylic acid, to acidify and trigger the gelation of Boc-L-Dopa(Bn)_2_-OH solutions. Our findings showed that by varying the mixing ratios of these two molecules, we could effectively fine-tune both the mechanical properties and the final pH of the gels [32].

**Figure 1:**
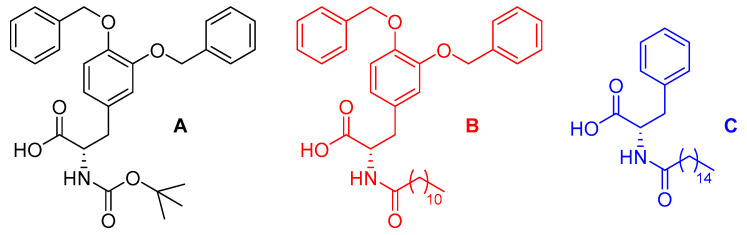
Chemical structure of the gelators: Boc-Dopa(Bn)_2_-OH (**A**) (black), Lau-Dopa(Bn)_2_-OH (**B**) (red), and Pal-Phe-OH (**C**) (blue).

In this paper, we report our recent findings regarding the use of a single acetylated amino acid, acetyl-L-valine (Ac-Val), as a pH modifier. We tested its efficacy as a trigger on three different amino acid derivatives that behave as efficient gelators when submitted to the pH change method: Boc-Dopa(Bn)_2_-OH (Figure 1A) [33], Lau-Dopa(Bn)_2_-OH (Figure 1B) [34], and Pal-Phe-OH (Figure 1C) [35,36]. It has been previously reported that gelators A and C are fully biocompatible [27,37] and can be used to prepare materials for drug delivery or tissue engineering. We also included gelator B in our study, as it is very similar to the others, so it is reasonable to think that it is biocompatible too.

These three molecules contain aromatic rings, one stereogenic center, one carboxylic group, and one lipophilic side chain (Figure 1) and form materials that differ in terms of transparency, robustness, and elasticity. In addition, Pal-Phe-OH **C** is a supergelator, able to form gel in concentrations down to 0.025% *w/v,* while Boc-L-Dopa(Bn)_2_-OH A and Lau-Dopa(Bn)_2_-OH B form gels in the 0.3–0.5% *w/v* concentration range. In light of that, we studied the impact of the pH modifier, always at 0.5% *w/v* gelator concentration, to better compare the results. The gelator concentration may be increased to 1.0 or 2.0% *w/v*, thus forming stiffer gels that may be required for some given applications. The formation of gels under these conditions follows the same procedures here described for gels prepared with gelators in 0.5% *w/v* concentration.

The use of efficient and biocompatible gelators coupled with a reliable trigger is an interesting approach to forming materials for drug release that can be highly tuned, simply modifying the nature and/or the concentration of the gelator, the trigger, and the final pH [1,38,39,40,41,42]. This strategy is a powerful means to achieve site-specific and sustained release over long time periods. For this reason, it is of paramount importance to use a trigger that homogeneously modifies the solution pH over time, reaching the predetermined final value required by the system.

## 2. Results

Ac-Val is a cheap compound that may be purchased by several companies as a solid and is fully water-soluble. Alternatively, it can be easily synthesized and purified on a multigram scale. For comparative purposes, we also evaluated the use of a commercial lactic acid (LA) solution. In contrast with Ac-Val, commercial LA solutions typically lack a clearly defined concentration. This aspect can affect the reproducibility of the gel formation. In addition, being water-soluble, Ac-Val can be added as an aqueous solution. This solves the problem of slow dissolution during gel formation, making it a reliable and reproducible pH modifier. Although the price of Ac-Val is presently two to three times that of LA, Ac-Val offers the advantage of being pure, which LA is not. Additionally, buying Ac-Val in bulk would considerably lower its cost.

As an initial investigation into the suitability of Ac-Val as an effective pH modifier, we examined the gelation of the three gelators using 1.3 equivalents of GdL, LA, and Ac-Val as triggers. This specific amount was chosen based on our prior research, where 1.3 equivalents of GdL were predominantly employed.

To prepare the gels, the gelator was suspended in Milli-Q^®^ H_2_O, and one equivalent of NaOH (0.1 M aqueous solution) was added. The resulting solution was stirred and sonicated until the complete dissolution of the gelator was achieved. Subsequently, the trigger was introduced: Ac-Val and LA were added as aqueous solutions, while GdL was added in its solid form. In all cases, the final volume was adjusted to yield a 0.5% *w/v* concentration of the gelator. Following the addition of the trigger, the solutions were gently stirred for a few seconds and then left undisturbed for 16 h.

**Figure 2:**
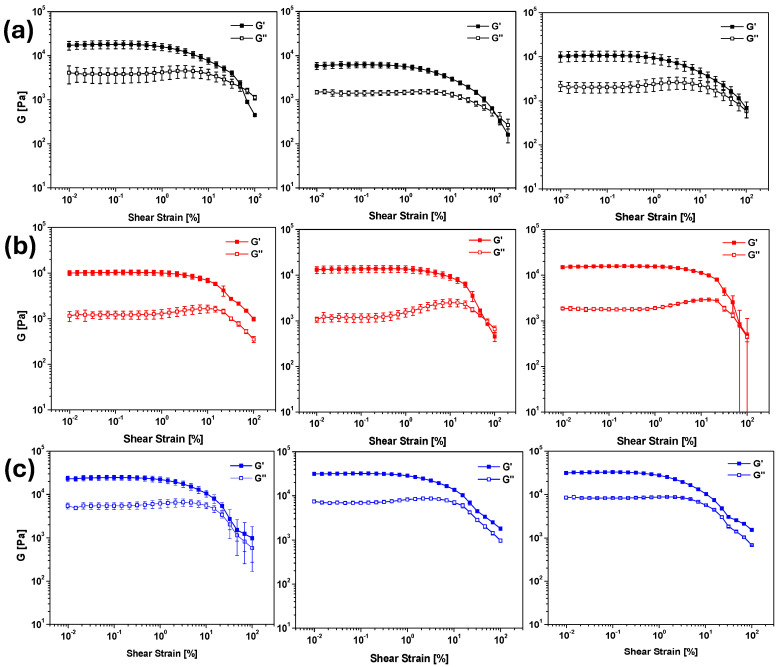
From top to bottom: results for amplitude sweep tests of the hydrogels of Boc-Dopa(Bn)_2_-OH (**a**) (black), Lau-Dopa(Bn)_2_-OH (**b**) (red), and Pal-Phe-OH (**c**) (blue), triggered with GdL (**left**), LA (**middle**), and Ac-Val (**right**), always 1.3 equiv. The experiments were repeated in triplicate, and results are expressed as mean ± standard deviation.

As anticipated, all the samples successfully formed gels (as depicted in Figure S1), and their mechanical properties were subsequently analyzed and compared using rheological analysis. The corresponding results are presented in Figure 2.

Comparison of the amplitude sweeps revealed that the gels formed using the three triggers exhibited comparable strength, with storage moduli (G’) ranging from 10^4^ to 10^5^ Pascal. A confirmation of these results has been obtained by the analysis of the gelation time of the nine samples by recording the time sweep analysis of their formation during the first 2 h (Figure S2).

These results seem to indicate that Ac-Val allows the formation of gels comparable to the GdL ones. A key benefit of Ac-Val is the ease of precise dosing, as it can be added as a solution at the desired concentration. In contrast, the use of LA presented notable challenges, as foreseen. The lack of a clearly defined concentration in the commercial samples required several repetitions of the experiments to achieve the desired pH range of 4.5 to 5 to obtain hydrogels comparable with the samples obtained by adding GdL or Ac-Val.

**Figure 3:**
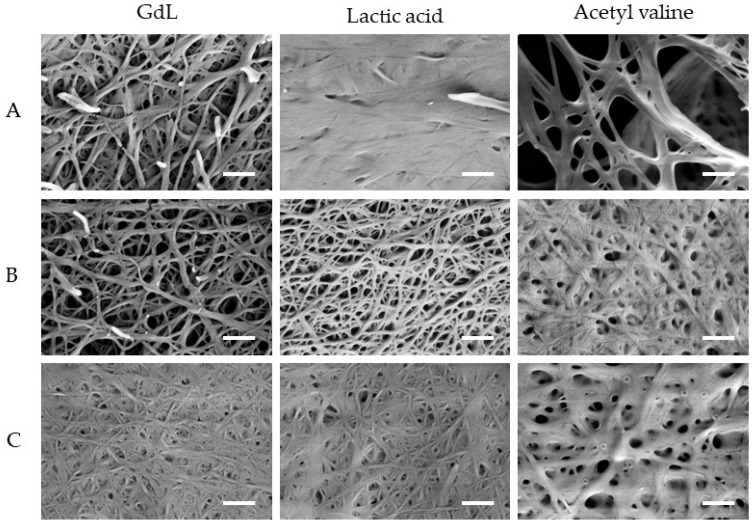
Scanning electron microscopy images of the aerogels of Boc-Dopa(Bn)_2_-OH (**A**) (**top**), Lau-Dopa(Bn)_2_-OH (**B**) (**middle**), and Pal-Phe-OH (**C**) (**bottom**), triggered with GdL (**left**), LA (**middle**), and Ac-Val (**right**), always using 1.3 equiv. Scale bar: 600 nm.

To better check the properties of the materials, we prepared the corresponding aerogels by freeze-drying the sample, and we analyzed the samples by SEM (Figure 3).

The SEM analysis showed a fibrillar morphology being present in all samples. Among the gelators, gelator A forms thicker fibrils with a lower interconnectivity, which correlates with the poorer rheological properties of these gels. On the other hand, gelators B and C form fibrils with comparable size and interconnectivity, with a higher propensity for gelator B to form a porous network.

Observing the different gels formed using different triggers, gelator A always shows fibrils of comparable size with no relevant amorphous materials observed covering the fibrils. Gelator B shows a maximum fibrillar thickness between 70 and 90 nm with all the triggers tested, with a small amount of less structurally defined material being observed covering the fibrils prepared using Ac-Val as a trigger. Finally, gelator C shows comparable fibrils when triggered with GdL or LA, both exhibiting clear fibrils with similar maximum thickness (49 ± 8 and 50 ± 10 nm, respectively, *N* = 10). On the other hand, when triggered using Ac-Val, the gelator forms thicker fibrils (with a maximum thickness of 120 ± 30 nm, *N* = 10) while also exhibiting a less structured phase covering the fibrils. It has to be taken into account that the higher thickness observed may be an artifact due to the amorphous phase deposited on the fibrils.

The samples have been analyzed also by ATR-FTIR spectroscopy (Figure S3), and we could not detect any substantial difference among the absorption bands.

Freeze-dried gels were also analyzed using X-ray powder diffraction (XRPD) to evaluate their crystal structure (see Figures S4–S6). All samples showed a crystal pattern, with a different one for each of the three gelators. Despite that, all the gelators showed the most intense diffraction peak at a low angle, corresponding to about 18 Å for gelator A, 29 Å for gelator B, and 32 Å for gelator C. Each of these lengths is comparable with the length of the gelators’ molecule fully extended.

Gelator A (Boc-Dopa(Bn)_2_-OH, Figure S4) showed no relevant crystallographic differences when triggered using LA or Ac-Val but exhibits a completely different crystal structure when triggered using GdL. This last pattern shows a split of the main peak, located around 4.8° (18.4 Å) in the LA and Ac-Val triggered gels, into two diffraction peaks at 4.7° (18.8 Å) and 5.1° (17.1 Å), respectively. This split suggests this polymorphic form presents two different conformations of the gelator, one more extended and one more compact, while the LA and Ac-Val crystal form has mostly a single conformation.

On the contrary, the gel obtained using gelator B (Lau-Dopa(Bn)_2_-OH, Figure S5) shows no relevant difference in crystal structure when prepared using the different triggers.

The gels prepared using gelator C (Pal-Phe-OH, Figure S6) also show similar diffraction patterns. Nonetheless, while the two gels prepared using GdL and Ac-Val show a similar level of crystallinity, a higher crystallinity was observed when LA is used. Using the Scherrer equation, it was possible to calculate the crystallite size on the main peak, corresponding to 29.4 nm for the gel prepared using LA and 15.6 nm for the gels prepared using GdL and Ac-Val. When observing the main peak, a small shift is also visible between samples. The sample prepared using LA is the one with the higher angle, 2.92° (30.0 Å), followed by the one prepared using Ac-Val, 2.83° (31.1 Å), and the one prepared using GdL, 2.70° (32.7 Å). This trend suggests how the three triggers induce a different average extension of the molecule, with the one triggered using GdL being the one with the higher extension. Other small shifts with the same trend are visible in the diffraction patterns as reported in Figure S6.

From the comparison of the results obtained using the three different triggers, we can conclude that Ac-Val is a valid trigger for forming gels via the pH change method with the three gelators.

**Figure 4:**
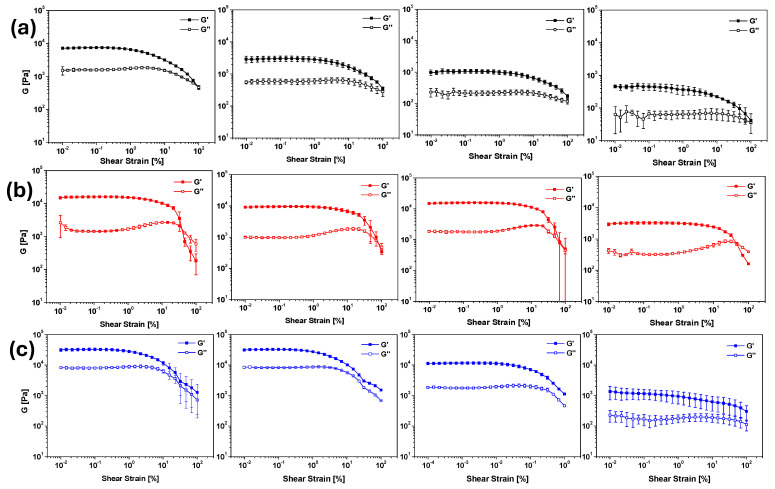
From top to bottom: results for amplitude sweep tests of the hydrogels of Boc-Dopa(Bn)_2_-OH (**a**) (black), Lau-Dopa(Bn)_2_-OH (**b**) (red), and Pal-Phe-OH (**c**) (blue), triggered with Ac-Val: from left to right 1.15 equiv., 1.00 equiv., 0.85 equiv., and 0.70 equiv. The experiments were repeated in triplicate, and results are expressed as mean ± standard deviation.

To obtain stable gels at neutral pH for drug delivery applications, we prepared gels with the three gelators, adding decreasing amounts of trigger to increase the final pH of the gels. The gelator was suspended in Milli-Q^®^ H_2_O, and NaOH (1 equivalent, 0.1 M aqueous solution) was added. The solution was stirred and sonicated until the gelator was completely dissolved. Then, Ac-Val was added as an aqueous solution. The final volume of each sample was adjusted to achieve a 0.5% *w/v* gelator concentration. Following the trigger addition, the solutions were gently stirred for a few seconds and then left to stand for 16 h. All twelve additional samples formed gels (Figure S7), and their mechanical properties were analyzed and compared using rheological analysis. The results are shown in Figure 4.

**Figure 5:**
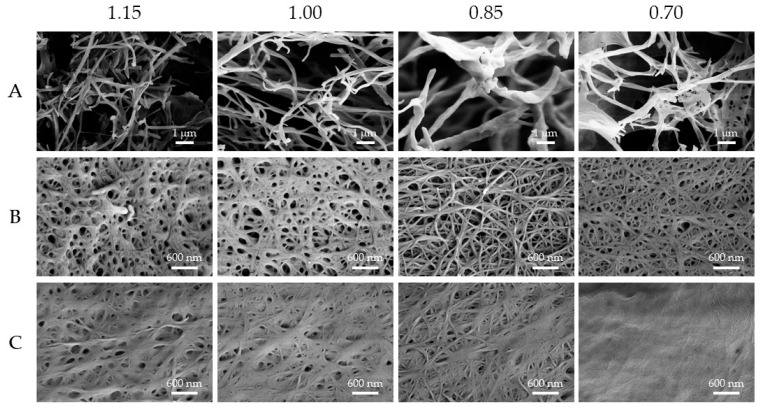
Scanning electron microscopy images of the aerogels of Boc-Dopa(Bn)_2_-OH (**A**) (**top**), Lau-Dopa(Bn)_2_-OH (**B**) (**middle**), and Pal-Phe-OH (**C**) (**bottom**), triggered with Ac-Val: from left to right 1.15 equiv., 1.00 equiv., 0.85 equiv., and 0.70 equiv.

The formation of a complex network that produces the gel formation was confirmed by SEM analysis of the corresponding aerogels (Figure 5). The results showed how decreasing the number of equivalents of Ac-Val used induces a fibrillar thickening and shortening in gelator A, leading to a lower interconnectivity and consequently a weaker gel. On the contrary, gelator B always shows a comparable fibrillar morphology with a gradual decrease in the amorphous phase covering the fibrils. Finally, gelator C shows comparable morphologies when using 1.15 and 1.00 equivalents and a slightly less compact phase when using 0.85 equivalents. Contrary to gelator B, a drastic morphological change is observed when using 0.70 equivalents, with fibrils barely visible and a continuous phase, made of small and compact fibers, being the main component of the hydrogel. For both gelators B and C, this decrease in the amorphous phase associated with the fibrils aligns with the decrease in the rheological properties of the gel. This may suggest that, when associated with a well-formed fibrillar network, the amorphous phase is a critical component of the hydrogel that may act as a binder between the fibrils, thus reinforcing the hydrogel network.

The samples were also analyzed using ATR-FTIR spectroscopy (Figure S8), and as expected, no significant differences were observed in the absorption bands.

The XRPD analyses of the samples highlighted small changes in the crystallographic structures when different equivalents of Ac-Val were used. The gelator A showed a shift of the main peak from 4.8° (18.3 Å) to 5.0° (17.8 Å) when moving from 1.00 eq. to 0.80 eq. of trigger. This may indicate how a higher pH may result in a more compact conformation of the gelator. A similar trend was observed for the peak at 9.6°, exhibiting a shift to 9.7°. Gelator B showed no shift in the main peak but one of a low-intensity signal from 14.9° to 13.9° when moving from 1.15 equivalents to 1.00 equivalents. Similarly, gelator C shows only a small shift of the signal from 4.0° to 4.2° when moving from 1.15 equivalents to 1.00 equivalents. It is interesting to notice how this shift brings the signal to the same position as when GdL is used. No relevant changes in the overall crystallinity or the main peak crystallite size were observed, decreasing the amount of trigger for any of the gelators.

Table 1 summarizes the results obtained for gel formation with the three gelators. In all cases gels were formed, although their strength decreased as the pH increased, approaching physiological values. We can thus confirm that Ac-Val provides a highly tuneable approach, allowing for precise control of the desired pH by adjusting the equivalents added to the gelator. Specifically, the addition of 0.7 equivalents consistently resulted in gels, close to physiological pH, suitable for drug-delivery applications. Furthermore, by simply adjusting the Ac-Val equivalents, gels with any pH value between 4 and 7 can be easily formed. Carefully tuning the conditions of gel formation and using higher concentrations of gelator (i.e., 1% or 2% *w/v*), it is not only possible to make gels with a final pH reaching 7.5–7.6 but also to obtain materials with higher G’ moduli.

**Figure 6:**
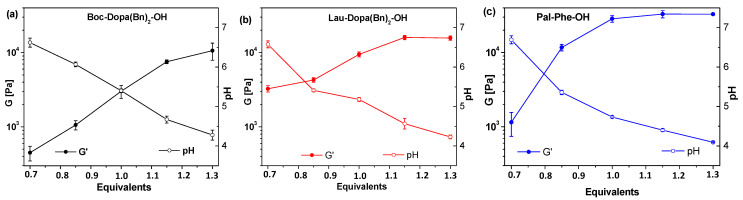
Correlation between G’ and pH of hydrogels of Boc-Dopa(Bn)_2_-OH (**a**) (black), Lau-Dopa(Bn)_2_-OH (**b**) (red), and Pal-Phe-OH (**c**) (blue) triggered with variable equivalents of Ac-Val.

To investigate whether the addition of less than one equivalent of the trigger could induce fibril formation, we conducted ^1^H NMR spectroscopy. This technique is highly informative [26,41,42] because molecules incorporated into fibers are no longer in solution and thus are not visible in the NMR spectrum. We acquired ^1^H NMR spectra of the three gels formed by adding Ac-Val (0.7 equiv.) to a solution of the gelator in D_2_O/NaOD. These spectra were recorded 16 h after the trigger addition, once the gels had set. For comparison, we also recorded ^1^H NMR spectra of Ac-Val in D_2_O and of the three gelators dissolved in D_2_O/NaOD before the trigger was introduced (Figures S12–S14). In all three hydrogel samples, the spectra displayed only the peaks corresponding to Ac-Val, indicating that this molecule does not participate in network formation. Conversely, the peaks associated with the gelators were absent, confirming their involvement in the network structure, even with only 0.7 equivalents of the trigger. This result suggests that the final pH is the primary determinant for fibril formation in gelators containing a carboxy group. Figure 6 presents a plot summarizing the strength of the hydrogels as a function of their pH, which can assist users in selecting the appropriate amount of Ac-Val to achieve the desired final pH.

The three samples exhibited gel-like behavior, as demonstrated by the rheological analysis presented in Figure 4 and summarized in Table 1.

To further characterize these promising materials, we evaluated their stability under heating (temperature sweep) and after shaking (thixotropy). Thixotropy is the property of a gel to become temporarily fluid when shaken or stirred (G” > G’), then they quickly recover the solid state (G’ > G”) [43,44].

**Figure 7:**
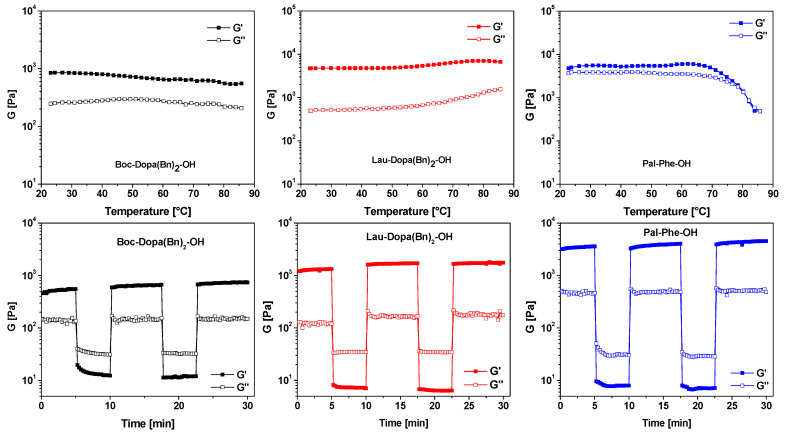
Top: results for temperature sweep tests of the hydrogels of Boc-Dopa(Bn)_2_-OH (black), Lau-Dopa(Bn)_2_-OH (red), and Pal-Phe-OH (blue), triggered with Ac-Val (0.70 equiv). Bottom: results for thixotropy tests of the hydrogels of Boc-Dopa(Bn)_2_-OH (black), Lau-Dopa(Bn)_2_-OH (red), and Pal-Phe-OH (blue), triggered with Ac-Val (0.70 equiv).

The results, shown in Figure 7, confirm that all three materials are both thixotropic and thermally stable. The gels formed with Boc-Dopa(Bn)_2_-OH (black) and Lau-Dopa(Bn)_2_-OH (red) remained stable up to 85 °C, while the gel derived from Pal-Phe-OH (blue) melted at approximately 80 °C. In all cases, these gels demonstrate sufficient stability for biomedical applications, as in drug delivery systems.

## 3. Materials and Methods

Chemicals—All chemicals and solvents were purchased from Sigma-Aldrich (Germany), apart from L-Dopa (TCI), palmitic acid (Acros Organics), and di-*tert*-butyl dicarbonate (Apollo Scientific). The purity of the reagents used is the following: L-Dopa (>98%), L-Phe-OMe·HCl (<98%), lauric acid (for synthesis), palmitic acid (98%), L-valine (99%), di-tert-butyl dicarbonate (99%), acetic anhydride (for synthesis), GdL (99%), and lactic acid (88%).

General Remarks for the Synthetic Procedure—All reactions were carried out in dried glassware, and all compounds were dried in vacuo.

The gelators A, B, and C were prepared according to the procedures described in previous papers [34,36,37]. All the characterization data matched the literature values.

The SEM analyses were acquired with a LEO 1530 FEG (Zeiss, Oberkochen, Germany) using a voltage of 5 kV, an aperture of 60 µm, and a working distance of 7 mm. Prior to analyses, the samples were frozen with liquid nitrogen and freeze-dried. Prior to analyses, a portion of the resulting aerogel was glued on carbon tape and coated with about 20 nm of gold.

High-quality infrared spectra (64 scans) were obtained at 2 cm^−1^ resolution with an ATR-FTIR Agilent (Santa Clara, CA, USA) Cary 630 FTIR spectrometer. An XS pH 8 PRO Basic pH meter (XS Instruments, Carpi (MO), Italy) equipped with an XS Sensor Standard T BNC was used to measure the samples’ pH. NMR spectra were recorded with a Varian (Palo Alto, CA, USA) Inova 600 spectrometer at 600 MHz (^1^H NMR).

XRPD analyses were performed using an Empyrean from Malvern Panalytical (Malvern, Worcestershire, United Kingdom), equipped with a rotating flat holder (with a rotation time of 2 s), using Cu Kα radiation generated at 40 kV and 40 mA (λ = 1.54056 Å). The diffractograms were collected between 2° and 30° using a step size of 0.04° and 400 s of counting time.

The rheological analyses were performed using an Anton Paar (Graz, Austria) MCR 102 rheometer. A cup and vane measuring system was used, setting a gap of 2.2 mm. The gel samples were prepared in 7 mL Sterilin Cups^®^ that fit in the rheometer and were left to rest for 16 h at room temperature before their use.

Oscillatory amplitude sweep experiments were performed at 23 °C using a Peltier control system, and the data points were collected (γ: 0.01–100%) using a constant angular frequency of 10 Hz. Time Sweep experiments were performed at 23 °C (controlled by an integrated Peltier system) using a constant shear strain (γ) of 0.5% and a constant angular frequency (ω) of 10 rad/s, collecting 1 point every minute. Temperature sweep analyses were performed on 2 mL gels prepared in glass vials that fit the rheometer, at a constant γ (0.2%) and ω (10 rad/s), by increasing the temperature using a linear ramp from 23 to 85 °C, with a rate of 3 °C/min.

Synthesis of Ac-Val—Ac-Val was synthesized using an acetylation procedure previously reported in the literature [45]. A suspension of L-valine (2.38 g, 20.3 mmol) and acetic anhydride (5.0 mL, 5.40 g, 52.9 mmol) in methanol (9.0 mL) was stirred under reflux for 6 h. After cooling to room temperature, all volatiles were removed under reduced pressure, and the crude reaction was washed twice with diethyl ether and twice with hexane under sonication. The resulting colorless solid was collected by filtration and dried in vacuo. Yield: 2.31 g (14.5 mmol, 71.5%), colorless solid. Mp: 166–168 °C. ^1^H NMR (400 MHz, MeOD) δ: 4.32 (1 H,d,J = 5.7 Hz, NHCH), 2.16 (1 H, dsept, J = 6.9, 5.6 Hz, CH(CH_3_)_2_), 2.01 (3 H, s, C(O)CH_3_), 0.97 (6 H, dd, J = 6.9, 2.7 Hz); ^13^C NMR (101 MHz, MeOD) δ: 174.9 (C), 173.5 (C), 59.1 (CH), 31.6 (CH), 22.3 (CH_3_), 19.5 (CH_3_), 18.3 (CH_3_). The data are consistent with the literature [46].

Procedure for Gel Preparation—The gels used for the rheological analysis were prepared in 7.0 mL Sterilin Cups^®^, using the following procedure: the gelator was suspended in Milli-Q^®^ H_2_O, and 1.0 equivalents of NaOH (0.1 M, aq) were added. The solution was stirred and sonicated until the complete dissolution of the gelator. To trigger the formation of the gel, three different triggers were used: Ac-Val, lactic acid, and glucono-δ-lactone (GdL). Ac-Val and lactic acid were added in volume, while GdL was added by weight, according to the gelator equivalents required to trigger the gel formation. Acetyl Valin solutions were prepared dissolving in 1 mL of Milli-Q^®^ H_2_O respectively 1.3, 1.15, 1.0, 0.85 and 0.7 equivalents. Lactic acid solutions were prepared by diluting a solution of 11.88 M. After the addition of the trigger, the solutions were gently stirred and then placed at rest for 18 h.

Procedure for Gel Preparation for ^1^H NMR Analysis—The gels used for the ^1^H NMR analysis were prepared using the following procedure: The gelator 0.5% *w*/*v* was suspended in D_2_O, and 1.0 equivalent of NaOD (0.1 M, aq) was added. The solution was stirred and sonicated until the complete dissolution of the gelator. Before adding the trigger, the ^1^H NMR spectra was acquired. To trigger the formation of the gel, a small volume of Ac-Val (0.7 equivalents) dissolved in D_2_O was added to the NMR tube containing the dissolved gelators. After the addition of the trigger, the NMR tube was placed at rest for 18 h, and then the ^1^H NMR spectra of the gel were acquired.

## 4. Conclusions

This study delineates the utility of Ac-Val as an innovative pH-responsive hydrogelation trigger. The efficacy of Ac-Val was tested with three gelators, allowing the formation of hydrogels with tunable pH values that make them good candidates for their use in drug delivery systems.

This molecule exhibits complete aqueous solubility and prolonged solution stability (weeks), facilitating the preparation of stock solutions across a broad concentration range. Its aqueous addition ensures immediate and homogeneous dispersion within the gelator solution, leading to rapid formation of macroscopically homogeneous gel networks. This may lead to the fabrication of hydrogels with tunable final pH values, a feature of significant value for applications requiring precise pH microenvironments, particularly within drug delivery systems.

Mechanistically, the carboxylic acid functionality of Ac-Val induces an instantaneous pH adjustment at the desired pH, as LA, in contrast with GdL, exhibits slow, progressive pH reduction during the gelation process. Although XRPD analyses of the aerogels suggest that the three triggers may induce different organization of the molecules in the fibril formation, the final gels show similar properties at the same pH. These findings validate Ac-Val as an alternative and valid acidic trigger to those reported so far for LMWGs.

In conclusion, this acid-catalyzed hydrogelation strategy demonstrates broad applicability, capable of inducing gelation across a diverse array of molecules possessing acidic moieties.

## Figures and Tables

**Table 1:** Summary of the properties of the gels obtained by varying the equivalents of trigger (Ac-Val) with the three different gelators, A (Boc-Dopa(Bn)_2_-OH), B (Lau-Dopa(Bn)_2_-OH), and C (Pal-Phe-OH).

Gelator	Trigger	Trigger Equiv.	G’ (KPa)	G” (KPa)	pH
A	GdL	1.3	18.14 ± 3.82	3.82 ± 1.44	4.39 ± 0.10
	LA	1.3	6.21 ± 0.93	1.41 ± 0.19	5.06 ± 0.03
	Ac-Val	1.3	10.64 ± 2.73	2.03 ± 0.53	4.28 ± 0.12
		1.15	7.54 ± 0.43	1.59 ± 0.13	4.67 ± 0.09
		1.00	3.02 ± 0.59	0.59 ± 0.11	5.40 ± 0.05
		0.85	1.06 ± 0.15	0.22 ± 0.05	6.07 ± 0.06
		0.70	0.45 ± 0.10	0.07 ± 0.05	6.62 ± 0.11
B	GdL	1.3	13.71 ± 1.28	1.18 ± 0.24	4.33 ± 0.04
	LA	1.3	10.35 ± 2.46	1.22 ± 0.25	4.48 ± 0.17
	Ac-Val	1.3	15.70 ± 1.01	1.79 ± 0.14	4.23 ± 0.05
		1.15	15.98 ± 1.06	1.44 ± 0.06	4.56 ± 0.13
		1.00	9.48 ± 0.73	0.96 ± 0.06	5.18 ± 0.05
		0.85	4.46 ± 0.30	0.42 ± 0.03	5.40 ± 0.04
		0.70	3.26 ± 0.31	0.32 ± 0.03	6.57 ± 0.09
C	GdL	1.3	24.54 ± 3.22	5.44 ± 0.95	4.15 ± 0.03
	LA	1.3	32.02 ± 1.96	6.89 ± 0.50	4.54 ± 0.05
	Ac-Val	1.3	32.87 ± 0.82	8.24 ± 0.26	4.09 ± 0.01
		1.15	32.97 ± 3.62	8.05 ± 0.73	4.40 ± 0.04
		1.00	28.50 ± 2.83	5.68 ± 0.79	4.73 ± 0.03
		0.85	11.71 ± 1.17	1.78 ± 0.14	5.36 ± 0.06
		0.70	1.15 ± 0.41	0.17 ± 0.70	6.69 ± 0.10

## Data Availability

The authors confirm that the data supporting the findings of this study are available within the article and its Supplementary Materials.

## Supplementary Materials

The following supporting information can be downloaded at: https://www.mdpi.com/article/10.3390/molecules30163345/s1. Figure S1. Photographs of gels triggered with 1.3 equiv. of GdL, LA and Ac-Val; Figure S2. Time sweep of gels triggered with 1.3 equiv. of GdL, LA and Ac-Val; Figure S3. ATR-IR spectra of aerogels obtained with gels triggered with 1.3 equiv. of GdL, LA and Ac-Val; Figure S4. XRPD analyses of Boc-Dopa(Bn)_2_-OH aerogels obtained with gels triggered with 1.3 equiv. of GdL, LA and Ac-Val; Figure S5. XRPD analyses of of Lau-Dopa(Bn)_2_-OH aerogels obtained with gels triggered with 1.3 equiv. of GdL, lactic acid and Ac-Val; Figure S6. XRPD analyses of Pal-Phe-OH aerogels obtained with gels triggered with 1.3 equiv. of GdL, LA and Ac-Val; Figure S7. Photographs of gels triggered with decreasing equivalents of Ac-Val; Figure S8. ATR-IR spectra of aerogels obtained with gels triggered with decreasing equivalents of Ac-Val; Figure S9. XRPD analyses of Boc-Dopa(Bn)_2_-OH aerogels obtained with different equivalents of acetyl valine; Figure S10. XRPD analyses of Lau-Dopa(Bn)_2_-OH aerogels obtained with different equivalents of acetyl valine; Figure S11. XRPD analyses of Pal-Phe-OH aerogels obtained with different equivalents of acetyl valine; Figure S12. ^1^H NMR spectra of hydrogels obtained from Boc-L-DOPA(Bn)_2_-OH triggered with Ac-Val (0.7 equiv.) in D_2_O; Figure S13. ^1^H NMR spectra of hydrogels obtained from Lau-L-DOPA(Bn)_2_-OH triggered with Ac-Val (0.7 equiv.) in D_2_O; Figure S14. ^1^H NMR spectra of hydrogels obtained from Pal-Phe-OH triggered with Ac-Val (0.7 equiv.) in D_2_O.

## Abbreviations

The following abbreviations are used in this manuscript:

Ac-Val	acetyl-L-valine
Phe	phenylalanine
Dopa	3,4-dihydroxyphenylalanine
Lau	lauroyl
Pal	palmitoyl
LMWGs	low-molecular-weight gelators
GdL	glucono-δ-lactone
Boc	tert-butyloxycarbonyl
SEM	Scanning electron microscopy
NMR	Nuclear Magnetic Resonance
LA	lactic acid
XRPD	X-ray powder diffraction
ATR-IR	attenuated total reflection infrared spectroscopy

## References

[1] Abraham B.L., Toriki E.S., Tucker N.J., Nilsson B.L. (2020). Electrostatic Interactions Regulate the Release of Small Molecules from Supramolecular Hydrogels. J. Mater. Chem. B.

[2] Gaohua L., Miao X., Dou L. (2021). Crosstalk of Physiological PH and Chemical PKa under the Umbrella of Physiologically Based Pharmacokinetic Modeling of Drug Absorption, Distribution, Metabolism, Excretion, and Toxicity. Expert Opin. Drug Metab. Toxicol..

[3] Fallingborg J. (1999). Intraluminal PH of the Human Gastrointestinal Tract. Dan. Med. Bull..

[4] Bogdanov A., Bogdanov A., Chubenko V., Volkov N., Moiseenko F., Moiseyenko V. (2022). Tumor Acidity: From Hallmark of Cancer to Target of Treatment. Front. Oncol..

[5] Bae J.H., Kim H.S. (2024). A PH-Responsive Protein Assembly through Clustering of a Charge-Tunable Single Amino Acid Repeat. ACS Appl. Mater. Interfaces.

[6] Harrison T.D., Ragogna P.J., Gillies E.R. (2018). Phosphonium Hydrogels for Controlled Release of Ionic Cargo. Chem. Commun..

[7] Giuri D., Cenciarelli F., Tomasini C. (2024). Low-Molecular-Weight Gels from Amino Acid and Peptide Derivatives for Controlled Release and Delivery. J. Pept. Sci..

[8] Mahmood T., Sarfraz R.M., Mahmood A., Salem-Bekhit M.M., Ijaz H., Zaman M., Akram M.R., Taha E.I., Sahu R.K., Benguerba Y. (2024). Preparation, In Vitro Characterization, and Evaluation of Polymeric PH-Responsive Hydrogels for Controlled Drug Release. ACS Omega.

[9] Awhida S., Draper E.R., McDonald T.O., Adams D.J. (2015). Probing Gelation Ability for a Library of Dipeptide Gelators. J. Colloid Interface Sci..

[10] Das T., Häring M., Haldar D., Díaz D. (2018). Phenylalanine and Derivatives as Versatile Low-Molecular-Weight Gelators: Design, Structure and Tailored Function. Biomater. Sci..

[11] Podder D., Chowdhury S.R., Nandi S.K., Haldar D. (2019). Tripeptide Based Super-Organogelators: Structure and Function. New J. Chem..

[12] Ravarino P., Domenico N.D., Barbalinardo M., Faccio D., Falini G., Giuri D., Tomasini C. (2022). Fluorine Effect in the Gelation Ability of Low Molecular. Gels.

[13] Morris K.L., Chen L., Rodger A., Adams D.J., Serpell L.C. (2015). Structural Determinants in a Library of Low Molecular Weight Gelators. Soft Matter.

[14] Cravotto G., Cintas P. (2009). Molecular Self-Assembly and Patterning Induced by Sound Waves. The Case of Gelation. Chem. Soc. Rev..

[15] Mahler A., Reches M., Rechter M., Cohen S., Gazit E. (2006). Rigid, Self-Assembled Hydrogel Composed of a Modified Aromatic Dipeptide. Adv. Mater..

[16] Chen L., McDonald T.O., Adams D.J. (2013). Salt-Induced Hydrogels from Functionalised-Dipeptides. RSC Adv..

[17] Yang Z., Liang G., Xu B. (2008). Enzymatic Hydrogelation of Small Molecules. Acc. Chem. Res..

[18] Maeda H. (2008). Anion-Responsive Supramolecular Gels. Chem. Eur. J..

[19] Tang C., Smith A.M., Collins R.F., Ulijn R.V., Saiani A. (2009). Fmoc-Diphenylalanine Self-Assembly Mechanism Induces Apparent PK a Shifts. Langmuir.

[20] Rizwan M., Yahya R., Hassan A., Yar M., Azzahari A.D., Selvanathan V., Sonsudin F., Abouloula C.N. (2017). PH Sensitive Hydrogels in Drug Delivery: Brief History, Properties, Swelling, and Release Mechanism, Material Selection and Applications. Polymers.

[21] Chen Y., Li X., Bai J., Shi F., Xu T., Gong Q., Yang Z. (2018). A Supramolecular Hydrogel for Spatial-Temporal Release of Auxin to Promote Plant Root Growth. Chem. Commun..

[22] Li W. (2020). Supramolecular Nanofiber-Reinforced Puerarin Hydrogels as Drug Carriers with Synergistic Controlled Release and Antibacterial Properties. J. Mater. Sci..

[23] Chen X., Liu Z. (2016). A PH-Responsive Hydrogel Based on a Tumor-Targeting Mesoporous Silica Nanocomposite for Sustained Cancer Labeling and Therapy. Macromol. Rapid Commun..

[24] Kang J.H., Turabee M.H., Lee D.S., Kwon Y.J., Ko Y.T. (2021). Temperature and PH-Responsive in Situ Hydrogels of Gelatin Derivatives to Prevent the Reoccurrence of Brain Tumor. Biomed. Pharmacother..

[25] Wang J.T.W., Rodrigo A.C., Patterson A.K., Hawkins K., Aly M.M.S., Sun J., Al Jamal K.T., Smith D.K. (2021). Enhanced Delivery of Neuroactive Drugs via Nasal Delivery with a Self-Healing Supramolecular Gel. Adv. Sci..

[26] Zhao C., Wang Y., Shi B., Li M., Yan W., Yang H. (2022). Tailoring Co-Assembly Loading of Doxorubicin in Solvent-Triggering Gel. J. Colloid Interface Sci..

[27] Di Filippo M.F., Giuri D., Marchiori G., Maglio M., Pagani S., Fini M., Tomasini C., Panzavolta S. (2022). Self-Assembling of Fibers inside an Injectable Calcium Phosphate Bone Cement: A Feasibility Study. Mater. Today Chem..

[28] Adams D.J., Butler M.F., Frith W.J., Kirkland M., Mullen L., Sanderson P. (2009). A New Method for Maintaining Homogeneity during Liquid-Hydrogel Transitions Using Low Molecular Weight Hydrogelators. Soft Matter.

[29] Draper E.R., Mears L.L.E., Castilla A.M., King S.M., McDonald T.O., Akhtar R., Adams D.J. (2015). Using the Hydrolysis of Anhydrides to Control Gel Properties and Homogeneity in PH-Triggered Gelation. RSC Adv..

[30] Sinthuvanich C., Nagy-Smith K.J., Walsh S.T.R., Schneider J.P. (2017). Triggered Formation of Anionic Hydrogels from Self-Assembling Acidic Peptide Amphiphiles. Macromolecules.

[31] Zanna N., Merlettini A., Tomasini C. (2016). Self-Healing Hydrogels Triggered by Amino Acids. Org. Chem. Front..

[32] Shariati Pour S.R., Oddis S., Barbalinardo M., Ravarino P., Cavallini M., Fiori J., Giuri D., Tomasini C. (2023). Delivery of Active Peptides by Self-Healing, Biocompatible and Supramolecular Hydrogels. Molecules.

[33] Gaucher A., Dutot L., Barbeau O., Hamchaoui W., Wakselman M., Mazaleyrat J.P. (2005). Synthesis of Terminally Protected (S)-Β3-H-DOPA by Arndt-Eistert Homologation: An Approach to Crowned β-Peptides. Tetrahedron Asymmetry.

[34] Cenciarelli F., Pieraccini S., Masiero S., Falini G., Giuri D., Tomasini C. (2024). Experimental Correlation between Apparent PKa and Gelation Propensity in Amphiphilic Hydrogelators Derived from L-Dopa. Biomacromolecules.

[35] Cenciarelli F., Giuri D., Pieraccini S., Masiero S., D’Agostino S., Tomasini C. (2025). Phenylalanine-Based Amphiphilic Self-Assembled Materials: Gels or Crystals?. Chem. Eur. J..

[36] Schwaller D., Yilmazer S., Carvalho A., Collin D., Mésini P.J. (2023). Impact of Polymorphism in Oleogels of N-Palmitoyl-l-Phenylalanine. Soft Matter.

[37] Mehra R.R., Tiwari P., Basu A., Duttkonar A. (2019). In Search of Bioinspired Hydrogels from Amphiphilic Peptides: A Template for Nanoparticle Stabilization for the Sustained Release of Anticancer Drugs. New J. Chem..

[38] Sutton S., Campbell N.L., Cooper A.I., Kirkland M., Frith W.J., Adams D.J. (2009). Controlled Release from Modified Amino Acid Hydrogels Governed by Molecular Size or Network Dynamics. Langmuir.

[39] Jagrosse M.L., Agredo P., Abraham B.L., Toriki E.S., Nilsson B.L. (2023). Supramolecular Phenylalanine-Derived Hydrogels for the Sustained Release of Functional Proteins. ACS Biomater. Sci. Eng..

[40] Liu H., Bi X., Wu Y., Pan M., Ma X., Mo L., Wang J., Li X. (2021). Cationic Self-Assembled Peptide-Based Molecular Hydrogels for Extended Ocular Drug Delivery. Acta Biomater..

[41] Escuder B., LLusar M., Miravet J.F. (2006). Insight on the NMR Study of Supramolecular Gels and Its Application to Monitor Molecular Recognition on Self-Assembled Fibers. J. Org. Chem..

[42] Shapiro Y.E. (2011). Structure and Dynamics of Hydrogels and Organogels: An NMR Spectroscopy Approach. Prog. Polym. Sci..

[43] Nikoumanesh E., Poling-Skutvik R. (2023). The Effect of Thixotropy on the Yield Transition in Reversible, Colloidal Gels. J. Chem. Phys..

[44] Ohsedo Y., Taniguchi M., Oono M., Saruhashi K., Watanabe H. (2015). Long-Chain Alkylamide-Derived Oil Gels: Mixing Induced Onset of Thixotropy and Application in Sustained Drug Release. New J. Chem..

[45] Wende R.C., Seitz A., Niedek D., Schuler S.M.M., Hofmann C., Becker J., Schreiner P.R. (2016). The Enantioselective Dakin–West Reaction. Angew. Chem..

[46] Flitcroft C.E., Jolliffe K.A., McErlean C.S.P. (2023). Late-Stage, Stereoretentive, and Site-Selective Modification of Synthetic Peptides by Using Photoredox Catalysis. Chem. Eur. J..

