# Supplementary material for: Simple pH-Triggered Control over Hydrogel Formation by Acetyl Valine
Source: Molecules. 2025 Aug 11;30(16):3345. doi: 10.3390/molecules30163345 (PMC12388472; doi:10.3390/molecules30163345)
Supplement: Supplementary file 1 [file molecules-30-03345-s001.zip › molecules-3776012-supplementary.pdf]

SUPPORTING INFORMATION

Simple pH-Triggered Control over Hydrogel Formation by Acetyl Valine

Roberta Stile, Devis Montroni, Demetra Giuri and Claudia Tomasini *

Dipartimento di Chimica Giacomo Ciamician, Università di Bologna, Via Piero Gobetti, 85, 40129 Bologna, Italy

**Figure S1.** Photographs of gels triggered with 1.3 equiv. of GdL, LA and Ac-Val	Page S2
**Figure S2.** Time sweep of gels triggered with 1.3 equiv. of GdL, LA and Ac-Val	Page S3
**Figure S3.** ATR-IR spectra of aerogels obtained with gels triggered with 1.3 equiv. of GdL, LA and Ac-Val	Page S4
**Figure S4.** XRPD analyses of Boc-Dopa(Bn)$_2$-OH aerogels obtained with gels triggered with 1.3 equiv. of GdL, LA and Ac-Val.	Page S5
**Figure S5.** XRPD analyses of of Lau-Dopa(Bn)$_2$-OH aerogels obtained with gels triggered with 1.3 equiv. of GdL, LA and Ac-Val.	Page S5
**Figure S6.** XRPD analyses of Pal-Phe-OH aerogels obtained with gels triggered with 1.3 equiv. of GdL, LA and Ac-Val.	Page S6
**Figure S7.** Photographs of gels triggered with decreasing equivalents of Ac-Val	Page S7
**Figure S8.** ATR-IR spectra of aerogels obtained with gels triggered with decreasing equivalents of Ac-Val	Page S8
**Figure S9.** XRPD analyses of Boc-Dopa(Bn)$_2$-OH aerogels obtained with different equivalents of acetyl valine.	Page S9
**Figure S10.** XRPD analyses of Lau-Dopa(Bn)$_2$-OH aerogels obtained with different equivalents of acetyl valine.	Page S9
**Figure S11.** XRPD analyses of Pal-Phe-OH aerogels obtained with different equivalents of acetyl valine.	Page S10
**Figure S12.** ^{1}H NMR spectra of hydrogels obtained from Boc-L-DOPA(Bn)$_2$-OH triggered with Ac-Val (0.7 equiv.) in D_2O	Page S11
**Figure S13.** ^{1}H NMR spectra of hydrogels obtained from Lau-L-DOPA(Bn)$_2$-OH triggered with Ac-Val (0.7 equiv.) in D_2O	Page S12
**Figure S14.** ^{1}H NMR spectra of hydrogels obtained from Pal-Phe-OH triggered with Ac-Val (0.7 equiv.) in D_2O	Page S13

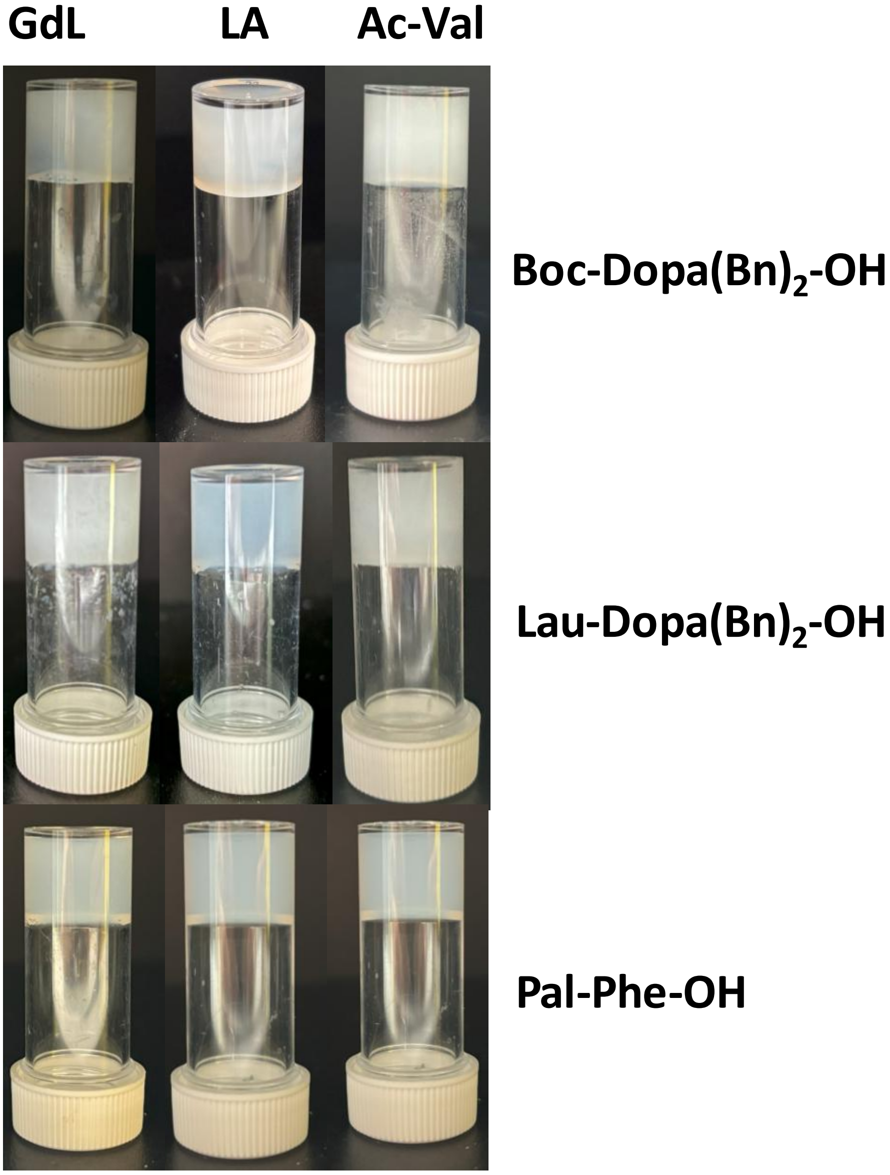

Figure S1. Photographs of gels triggered with 1.3 equiv. of GdL, LA and Ac-Val.

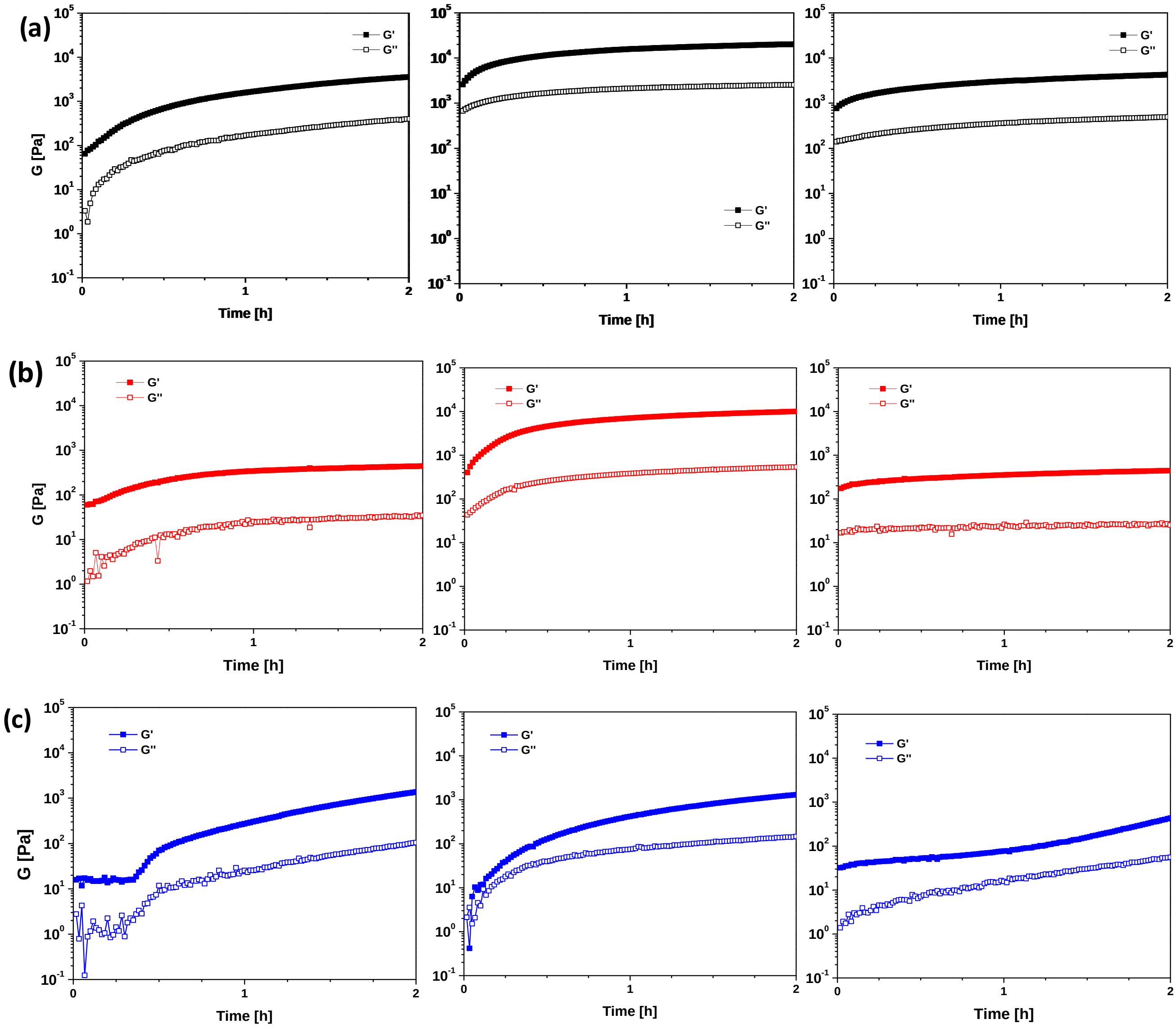

Figure S2. From top to bottom: results for time sweep tests of the hydrogels of Boc-Dopa(Bn)$_2$-OH **A** (black), Lau-Dopa(Bn)$_2$-OH **B** (red) and Pal-Phe-OH **C** (blue), triggered with GdL (left), LA (middle) and acetyl valine (right), always 1.3 equiv.

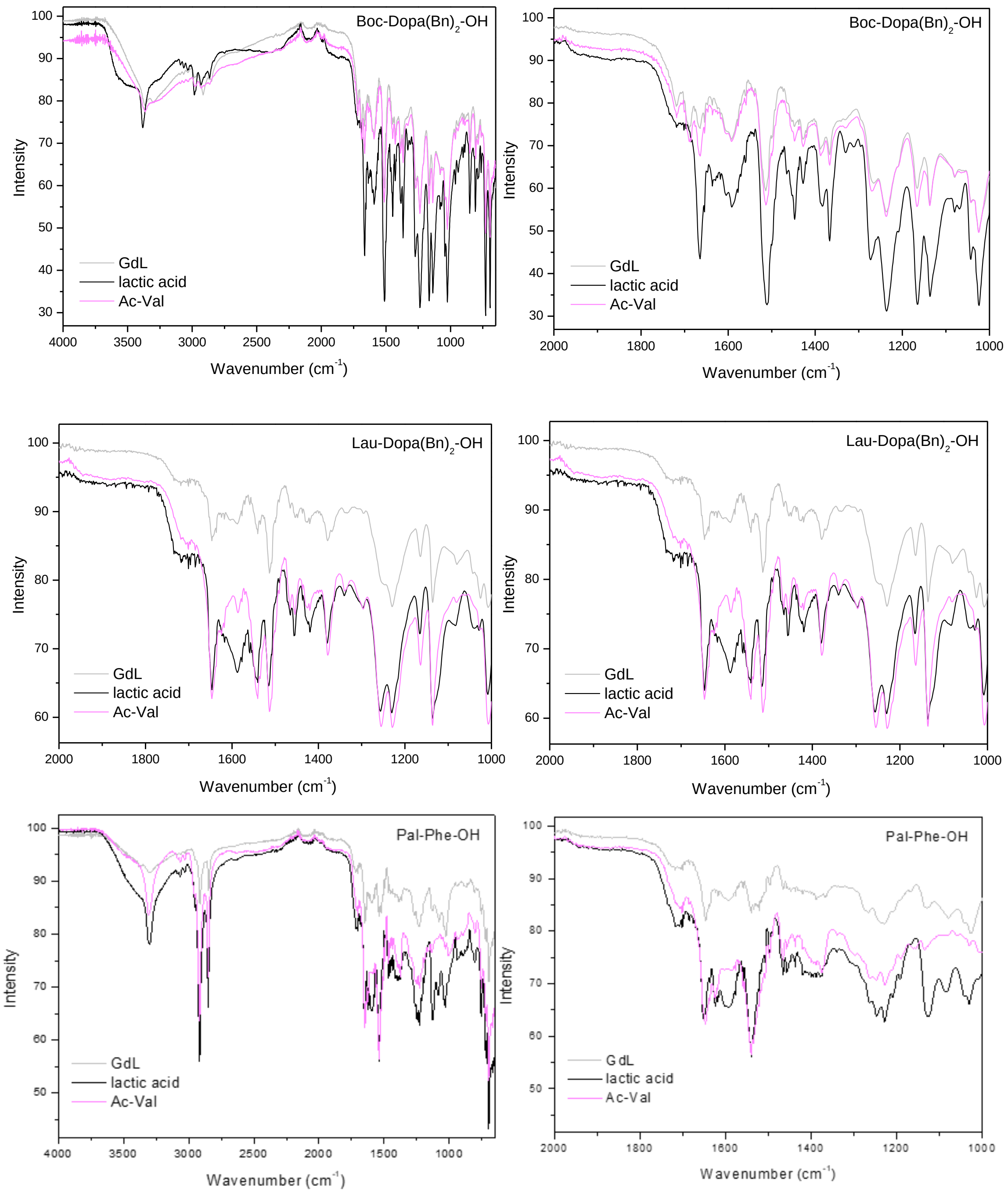

Figure S3. ATR-IR spectra of aerogels obtained with gels triggered with 1.3 equiv. of GdL (grey line), LA (black line) and Ac-Val (purple line). From to top to bottom: Boc-Dopa(Bn)$_2$-OH, Lau-Dopa(Bn)$_2$-OH, Pal-Phe-OH.

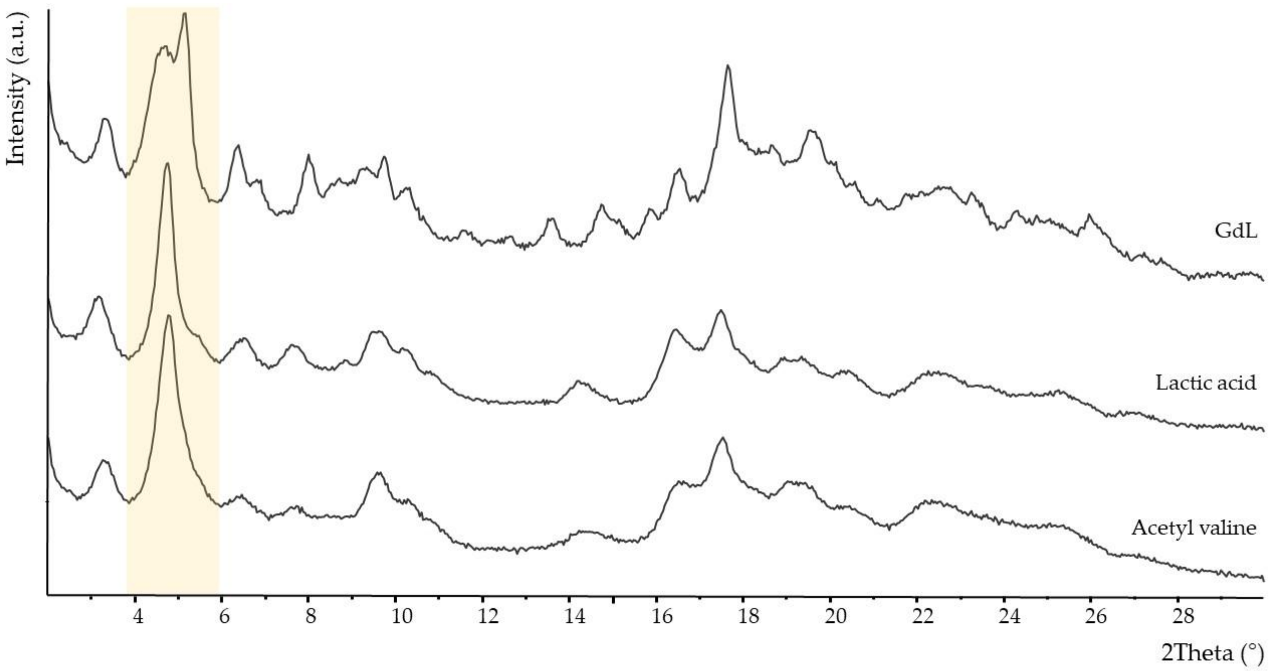

Figure S4. XRPD analyses of Boc-Dopa(Bn)$_2$-OH aerogels obtained with gels triggered with 1.3 equiv. of GdL (top), LA (middle) and Ac-Val (bottom). A yellow section was added to highlight the main diffraction peak.

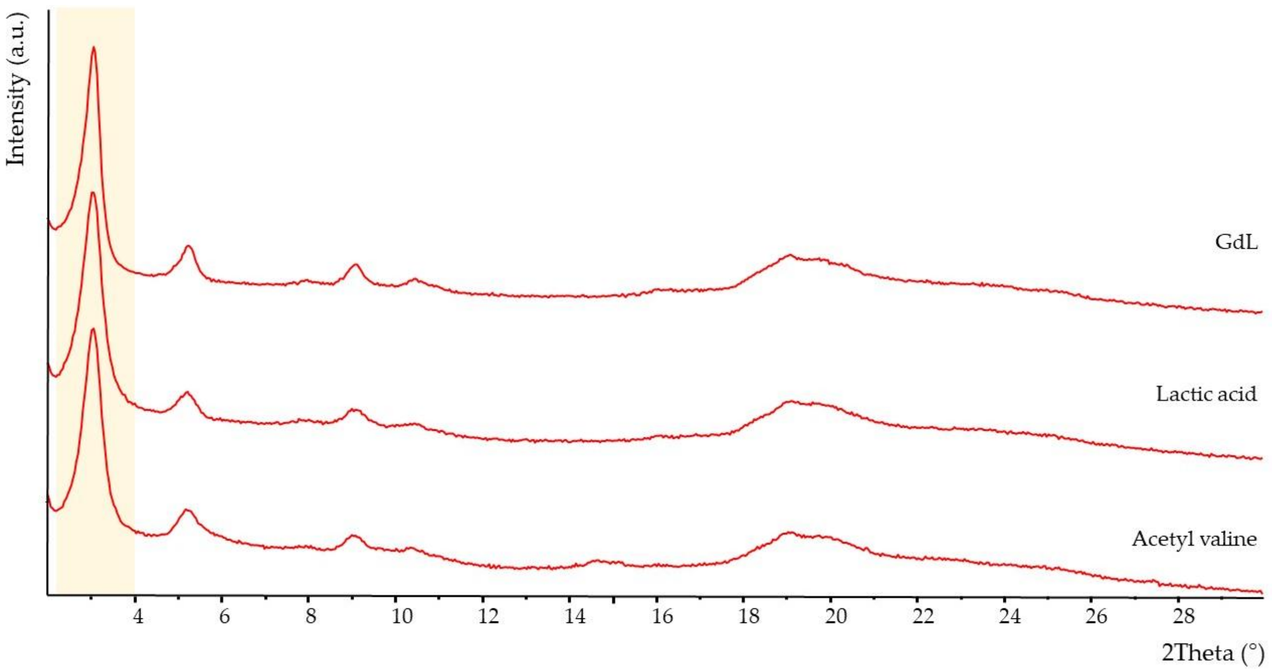

Figure S5. XRPD analyses of Lau-Dopa(Bn)$_2$-OH aerogels obtained with gels triggered with 1.3 equiv. of GdL (top), LA (middle) and Ac-Val (bottom). A yellow section was added to highlight the main diffraction peak.

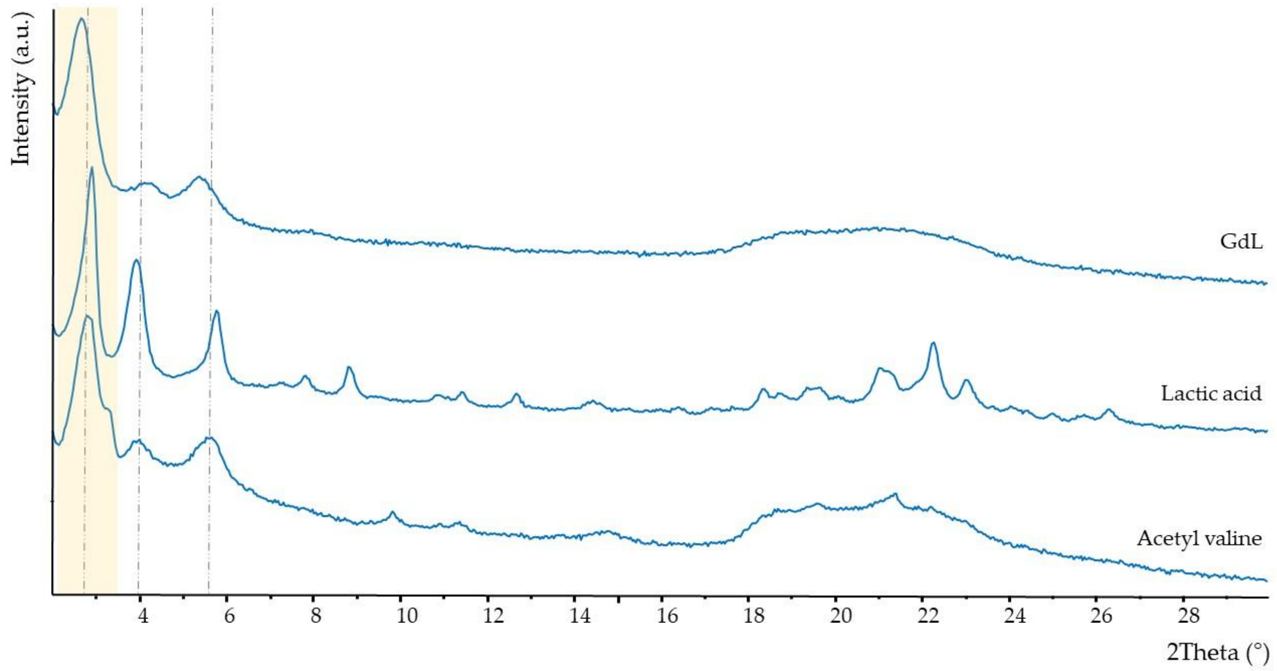

Figure S6. XRPD analyses of Pal-Phe-OH aerogels obtained with gels triggered with 1.3 equiv. of GdL (top), LA (middle) and Ac-Val (bottom). A yellow section was added to highlight the main diffraction peak while dashed lines indicate the peak position of the aerogel obtained using Ac-Val where shifts are observed.

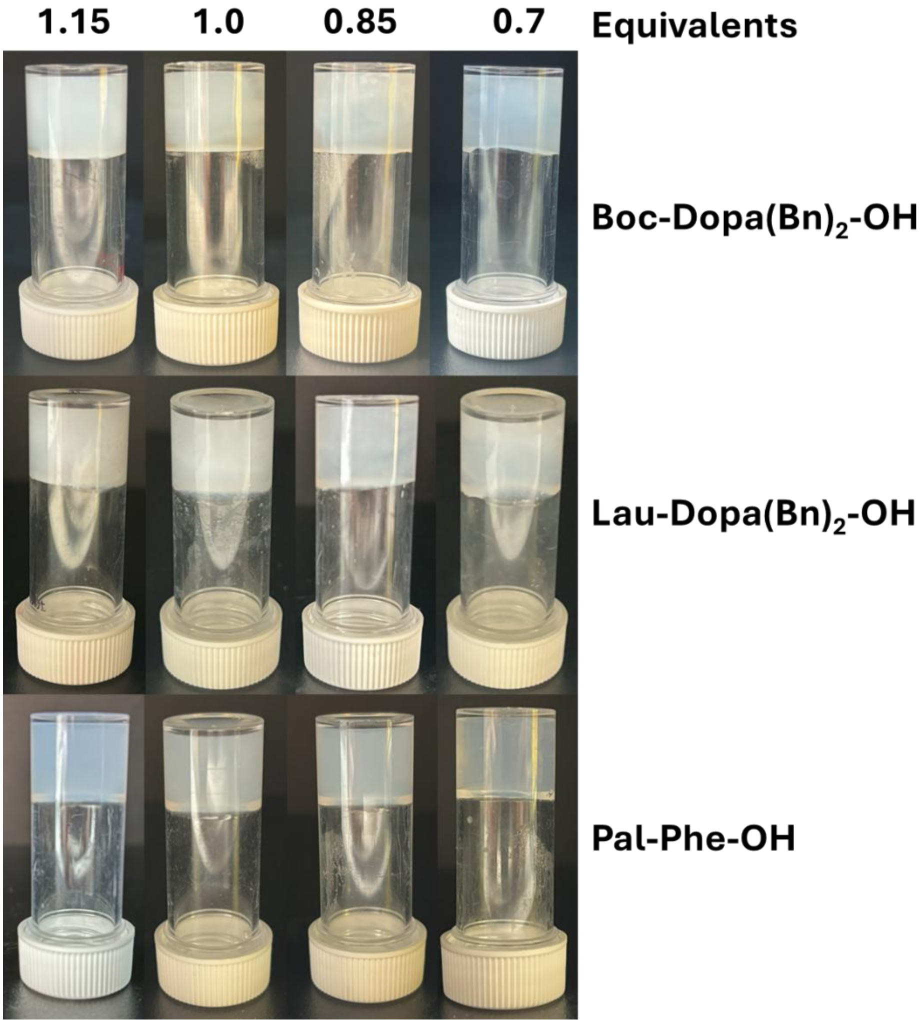

Figure S7. Photographs of gels triggered with decreasing equivalents of Ac-Val

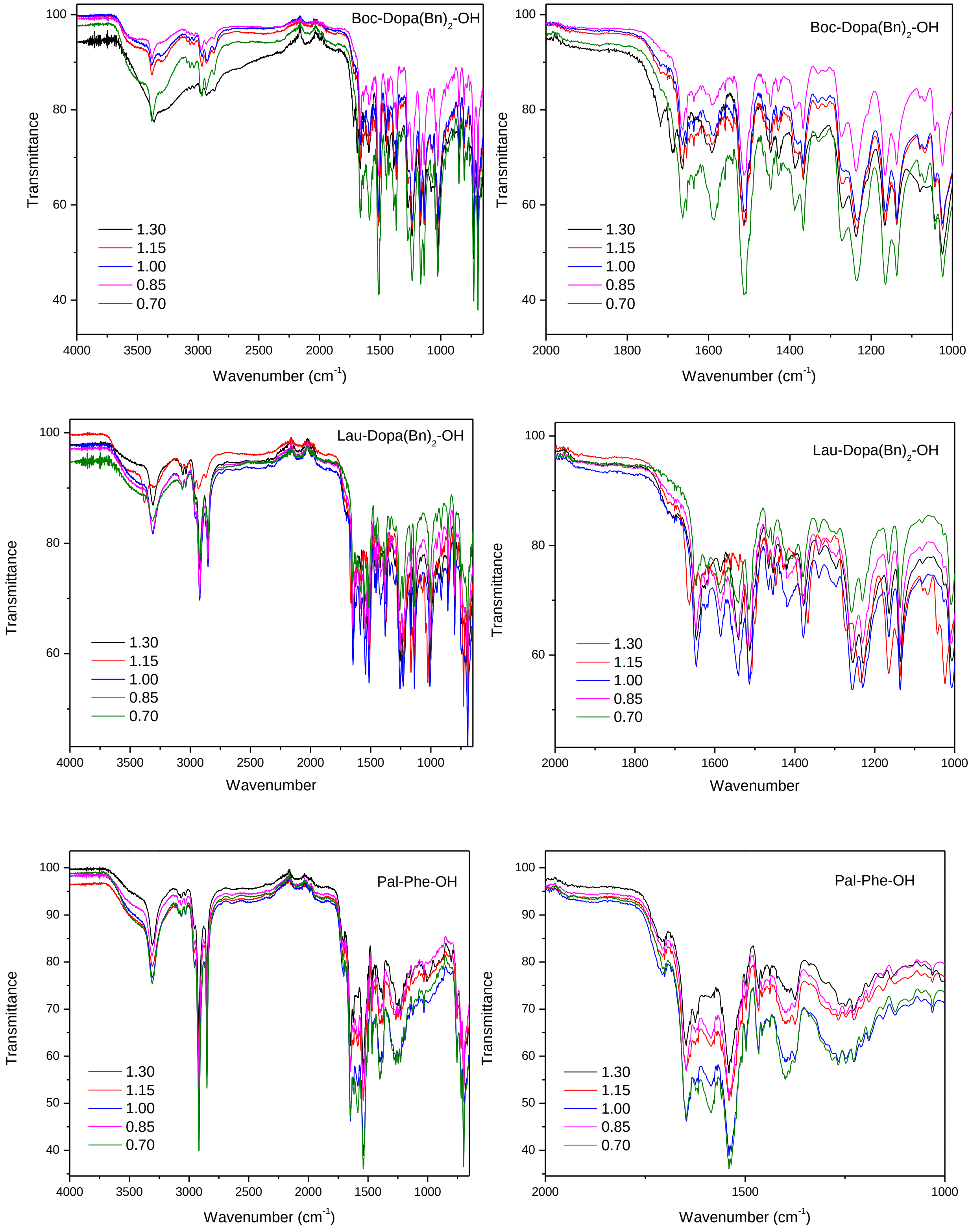

Figure S8. ATR-IR spectra of aerogels obtained with gels triggered with decreasing equivalents of Ac-Val. From to top to bottom: Boc-Dopa(Bn)$_2$-OH, Lau-Dopa(Bn)$_2$-OH, Pal-Phe-OH.

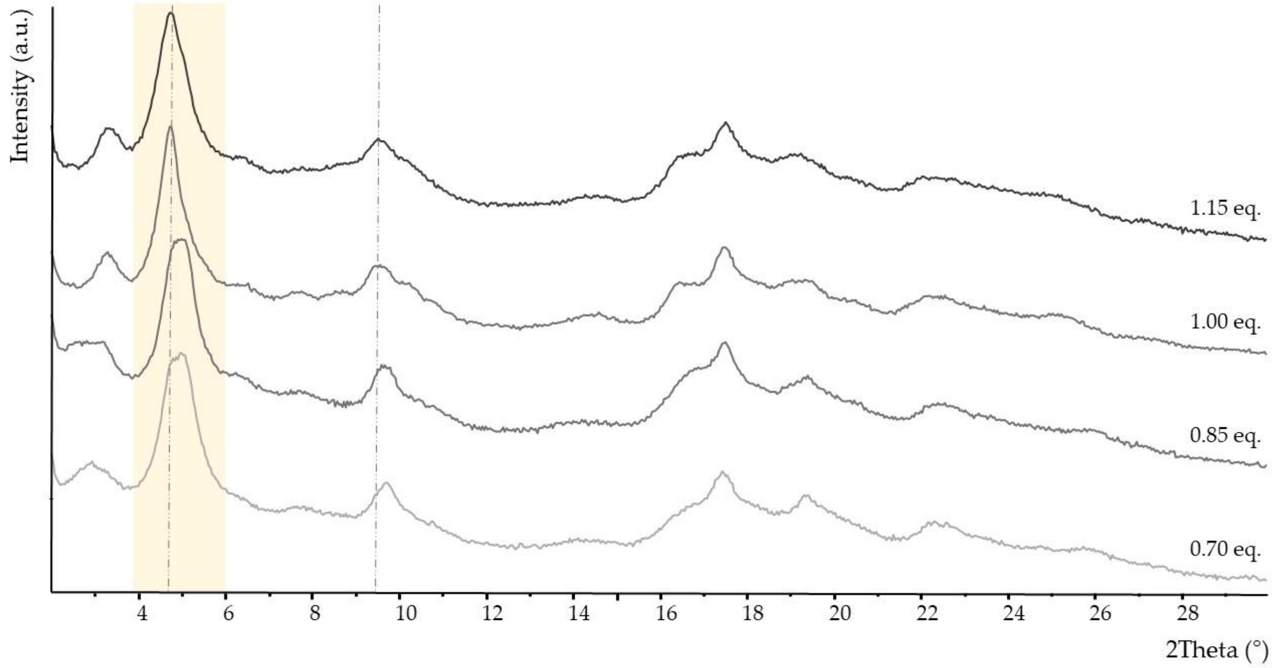

Figure S9. XRPD analyses of Boc-Dopa$(Bn)_2$-OH aerogels obtained with different equivalents of acetyl valine. From top to bottom: 1.15 equivalents, 1.00 equivalents, 0.85 equivalents, and 0.70 equivalents. A yellow section was added to highlight the main diffraction peak while dashed lines indicate the position of peaks of the aerogel obtained using 1.15 eq. where shifts are observed.

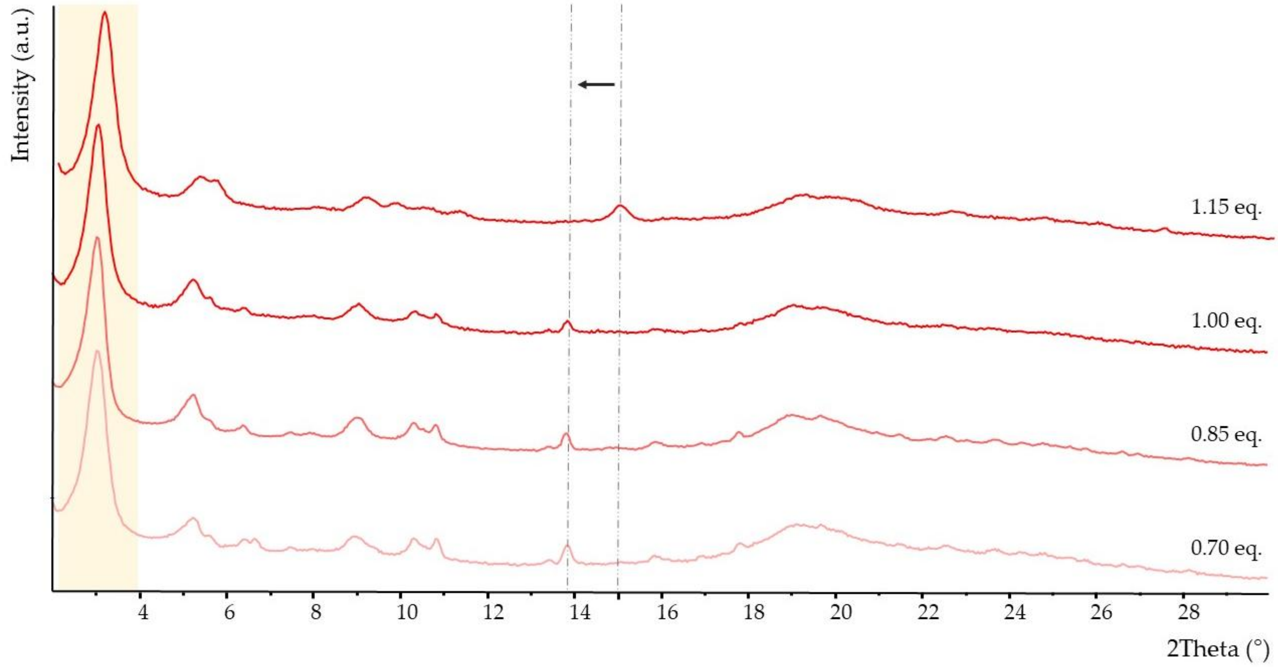

Figure S10. XRPD analyses of Lau-Dopa$(Bn)_2$-OH aerogels obtained with different equivalents of acetyl valine. From top to bottom: 1.15 equivalents, 1.00 equivalents, 0.85 equivalents, and 0.70 equivalents. A yellow section was added to highlight the main diffraction peak while dashed lines indicate the position of peaks of the aerogel obtained using 1.15 eq. where shifts are observed.

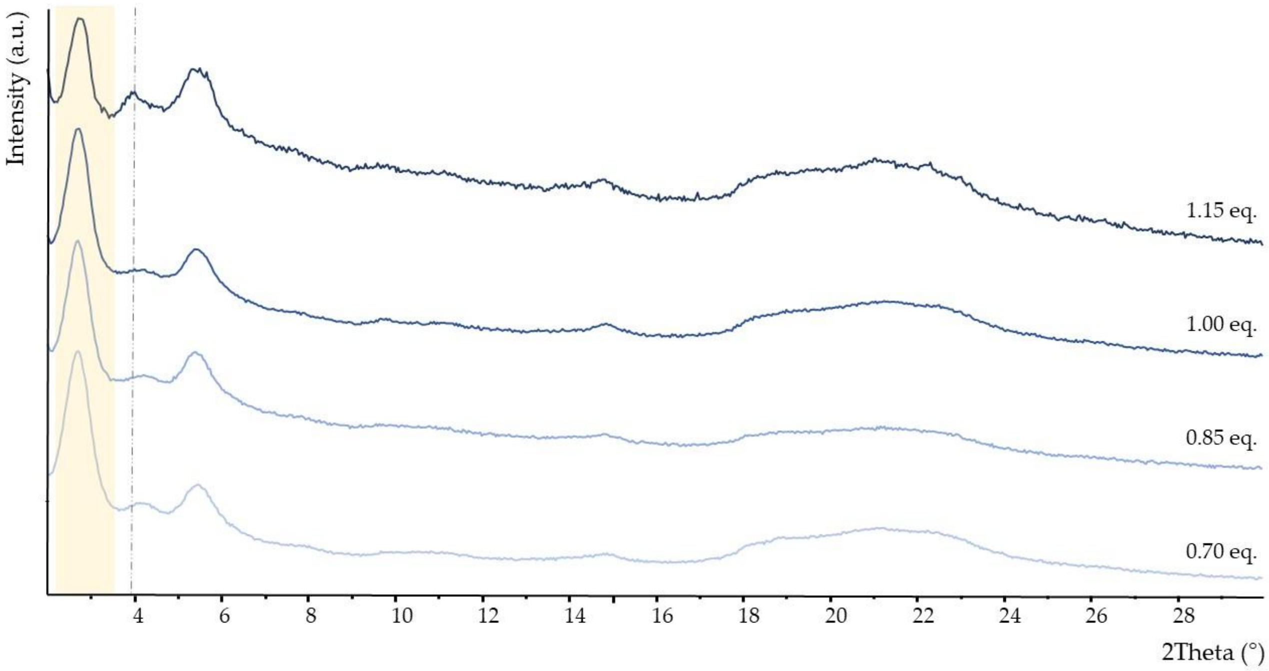

Figure S11. XRPD analyses of Pal-Phe-OH aerogels obtained with different equivalents of acetyl valine. From top to bottom: 1.15 equivalents, 1.00 equivalents, 0.85 equivalents, and 0.70 equivalents. A yellow section was added to highlight the main diffraction peak while dashed lines indicate the position of peaks of the aerogel obtained using 1.15 eq. where shifts are observed.

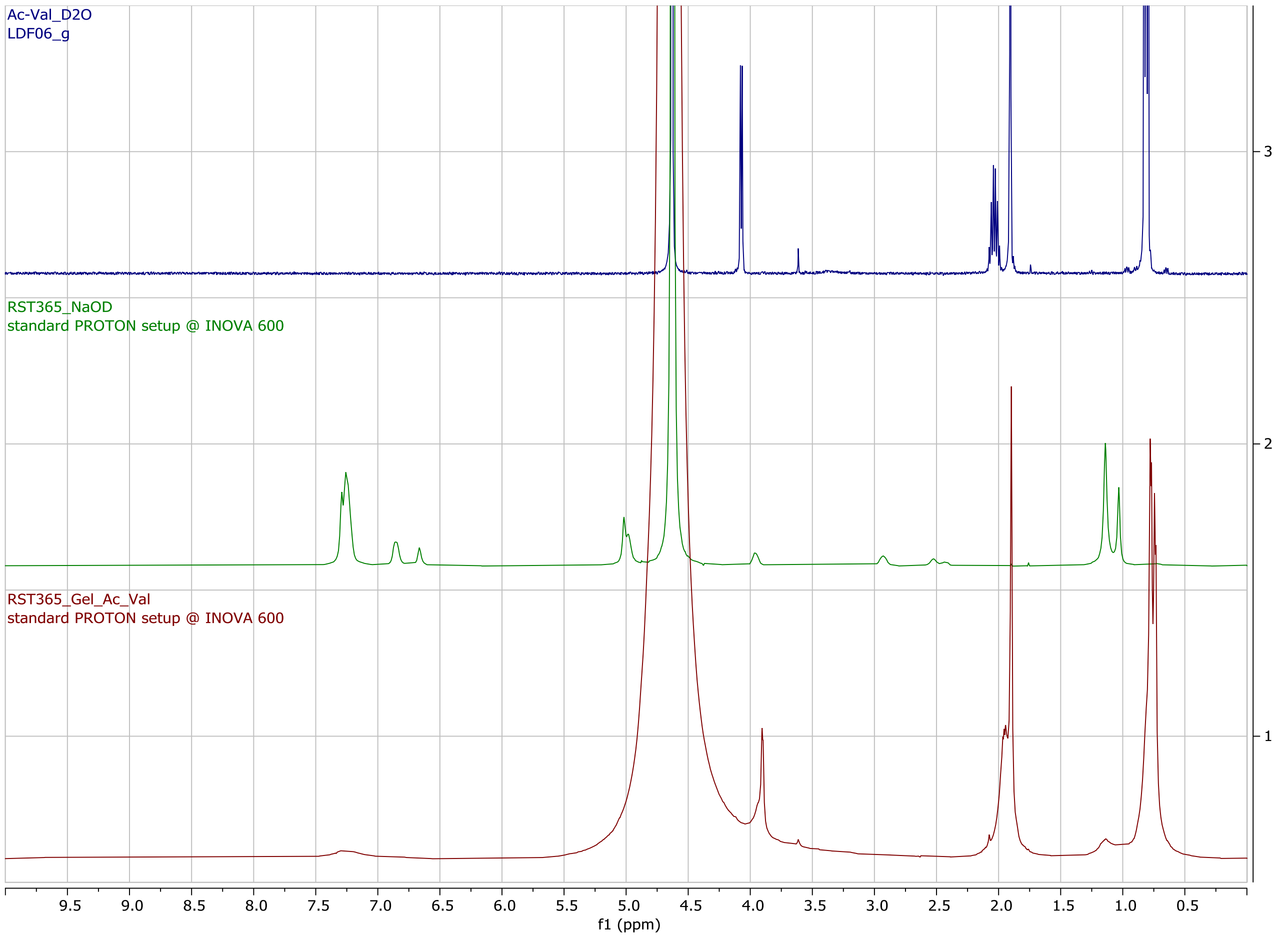

Figure S12. ^{1}H NMR spectra of hydrogels obtained from Boc-L-DOPA(Bn)$_2$-OH triggered with Ac-Val (0.7 equiv.) in D_2O. From top to bottom: solution of Ac-Val in D_2O; solution of Boc-DOPA(Bn)$_2$-OH in D_2O and NaOD; hydrogel obtained adding Ac-Val to the previous solution. The peaks of the gelator disappear while the peaks of Ac-Val are still visible, as the molecule is not involved in the network formation.

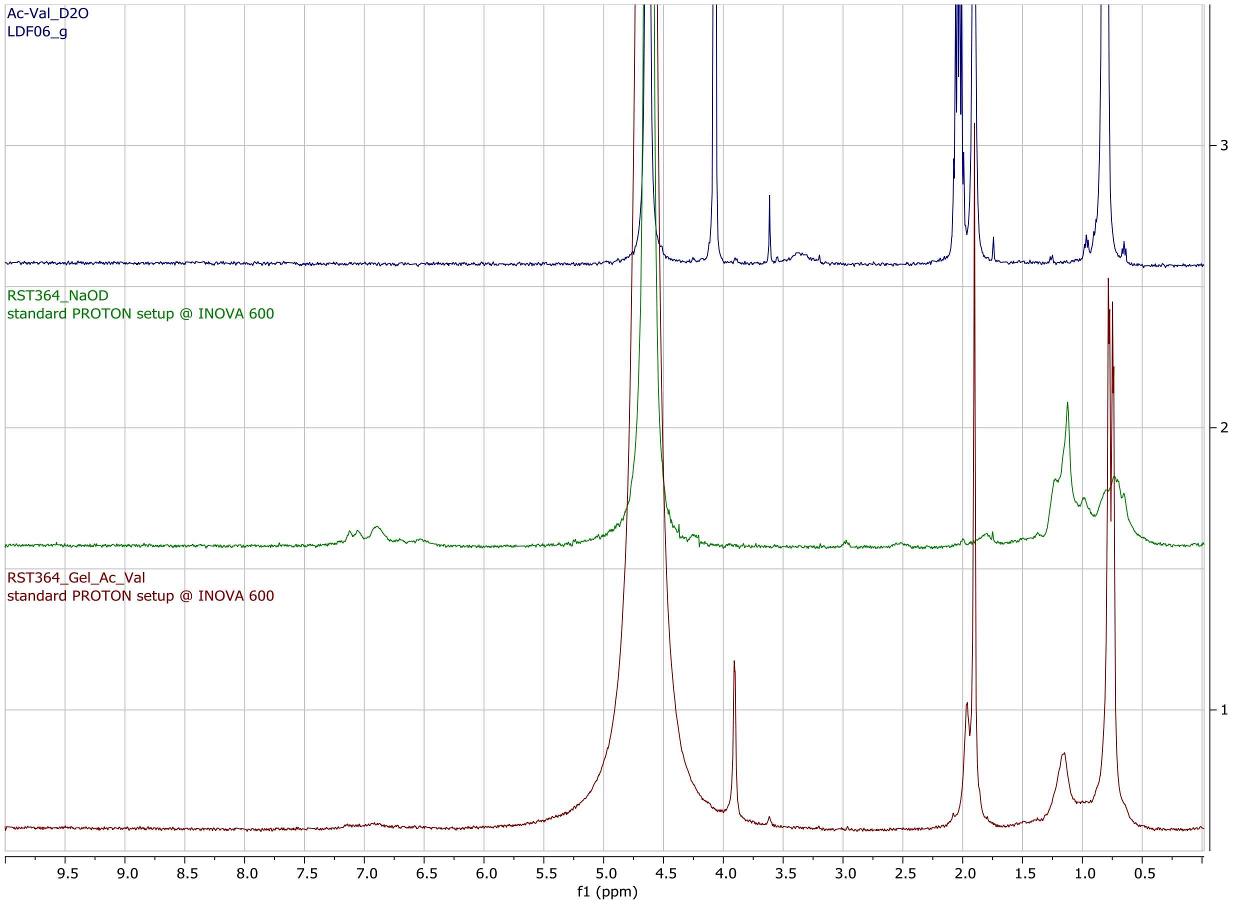

Figure S13. ^{1}H NMR spectra of hydrogels obtained from Lau-Dopa(Bn)$_2$-OH triggered with Ac-Val (0.7 equiv.) in D_2O. From top to bottom: solution of Ac-Val in D_2O; solution of Lau-Dopa(Bn)$_2$-OH in D_2O and NaOD; hydrogel obtained adding Ac-Val to the previous solution. The peaks of the gelator disappear while the peaks of Ac-Val are still visible, as the molecule is not involved in the network formation.

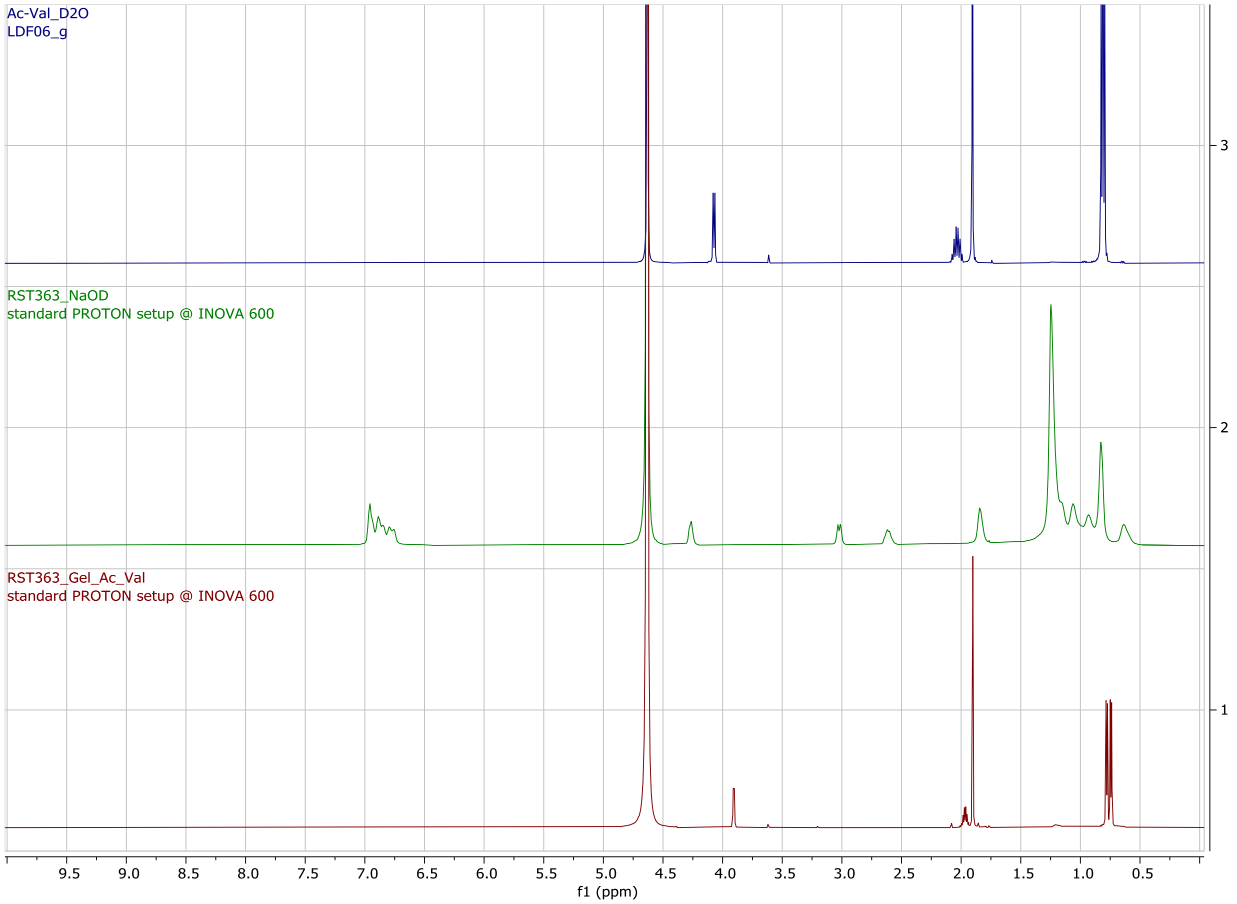

Figure S14. ^{1}H NMR spectra of hydrogels obtained from Pal-Phe-OH triggered with Ac-Val (0.7 equiv.) in D_2O. From top to bottom: solution of Ac-Val in D_2O; solution of Pal-Phe-OH in D_2O and NaOD; hydrogel obtained adding Ac-Val to the previous solution. The peaks of the gelator disappear while the peaks of Ac-Val are still visible, as the molecule is not involved in the network formation.
